# Digital Nutritional Care in Oncology: Opportunities to Enhance Quality of Life and Support Patient-Centered Care

**DOI:** 10.3390/cancers18152468

**Published:** 2026-07-31

**Authors:** Elisa Mattavelli, Valentina Da Prat, Lorenzo Perrone, Francesca De Simeis, Laura Boldrini, Salvatore Corallo, Annarita Sabbatini, Paolo Pedrazzoli, Roberto Biffi, Riccardo Caccialanza

**Affiliations:** 1 Department of Internal Medicine and Medical Therapy, University of Pavia, 27100 Pavia, Italy; e.mattavelli@smatteo.pv.it (E.M.);; 2 Clinical Nutrition and Dietetics Unit, Department of Oncology, Comprehensive Cancer Center, Fondazione IRCCS Policlinico San Matteo, 27100 Pavia, Italy; 3 Department of Oncology, Comprehensive Cancer Center, Fondazione IRCCS Policlinico San Matteo, 27100 Pavia, Italy; 4 Multidisciplinary Clinical Nutrition Service, European Institute of Oncology, IRCCS, 20141 Milan, Italy; 5 Division of Gynecology-Peritoneal Cancer Surgery Unit, Multidisciplinary Clinical Nutrition Service, European Institute of Oncology, IRCCS, 20141 Milan, Italy; 6 Department of Oncology and Hematology-Oncology, University of Milan, 20123 Milan, Italy

**Keywords:** digital tools, nutritional care, oncology, cancer care, quality of life, caregivers

## Abstract

**Background/Objectives**: Nutritional impairment is common across the cancer care continuum and may negatively affect treatment tolerance, functional status, quality of life, and caregiver burden. Digital tools may offer new opportunities to extend nutritional care beyond conventional clinical encounters, but their application in oncology remains heterogeneous and poorly defined. This narrative review aims to describe the current landscape of digital nutritional care in oncology, focusing on quality of life and patient perspectives while also considering the emerging role of caregivers. **Methods**: We conducted a narrative review of the available evidence on digital nutritional and lifestyle interventions in cancer care, including mobile applications, web-based platforms, teleconsultations, SMS- or email-based communication, remote monitoring systems, and hybrid models. **Results**: Digital nutritional and lifestyle interventions have been evaluated across active treatment, postoperative recovery, and survivorship. Available studies indicate that these interventions are generally feasible and may improve nutritional status, dietary behaviors, and physical activity levels. However, quality-of-life outcomes remain heterogeneous and have not been consistently assessed across studies. Some interventions combining digital tools with personalization, professional feedback, behavioral support, or clinically actionable monitoring reported favorable patient-centered outcomes. However, heterogeneity across studies limits the interpretation of these findings and the identification of specific intervention features associated with effectiveness. Patient and caregiver perspectives further highlight the importance of usability, trust, access to reliable information, communication with healthcare professionals, and practical support in the home setting. **Conclusions**: Digital nutritional care has the potential to extend and strengthen the delivery of nutritional care. Rather than replacing conventional interventions, it should be implemented through hybrid, supervised, equitable, and clinically integrated models. Future studies should consistently assess quality of life and other patient-reported outcomes while also addressing caregiver needs, digital burden, psychological safety, and inequalities in access.

## Simple Summary

Nutritional impairment is common among people with cancer and may negatively affect treatment tolerance, physical functioning, quality of life, and caregiver burden. Digital tools, including mobile applications, web-based platforms, teleconsultations, and remote monitoring systems, may help extend nutritional care beyond hospital visits and provide more continuous support in the home setting. This narrative review summarizes the current evidence on digital nutritional care in oncology, with particular attention to quality of life and patient perspectives, while also considering the emerging role of caregivers, implementation challenges, and potential risks. Available studies suggest that digital interventions are feasible and may improve nutritional status, dietary behaviors, weight-related outcomes, and physical activity. However, their effects on quality of life remain inconsistent, and this outcome has not been consistently assessed. Digital nutritional care has the potential to extend and strengthen the delivery of nutritional care. Rather than replacing conventional interventions, it should be implemented through supervised and clinically integrated models.

## 1. Introduction

Nutritional status is increasingly recognized as an important determinant of clinical outcomes throughout the cancer care continuum [1]. Both undernutrition and overnutrition may negatively influence prognosis, treatment tolerance, functional status, and quality of life [2]. Beyond body weight, nutritional impairment encompasses a complex spectrum of conditions including loss of muscle mass, sarcopenia, obesity, and sarcopenic obesity [3]. These alterations may result from the cancer itself, through tumour-related systemic inflammation and catabolic processes [4], as well as from reduced physical activity [5] and the nutritional effects of anticancer treatments, which may reduce dietary intake and/or nutrient absorption [6] or promote weight gain [7,8]. In this context, nutritional care should not be considered an ancillary intervention but rather an integral component of comprehensive cancer care [9,10]. Timely nutritional assessment and personalized nutritional interventions have demonstrated clinical benefits across the full spectrum of nutritional impairment. In patients with malnutrition or at risk of malnutrition, individualized nutritional support can improve nutritional status [11], survival [12,13], treatment tolerance [14,15], and quality of life [11]. Similarly, in patients with overnutrition, lifestyle intervention combining healthy nutritional counselling and physical activity may support weight management [16,17,18,19], reduce treatment-related symptoms [19], and improve quality of life [19,20]. Beyond active treatment, nutritional and lifestyle care remain important for supporting metabolic health and quality of life during long-term follow-up [21,22,23,24], while emerging evidence suggests a potential role in reducing cancer recurrence in selected survivor populations [25,26,27].

Effective nutritional care should also recognize caregivers as key partners in managing cancer-related nutritional issues. During active treatment and long-term follow-up, caregivers often support patients with meal planning, food preparation, monitoring dietary intake, managing nutrition impact symptoms, and interpreting dietary advice [28,29]. Despite growing recognition of the importance of nutritional care in oncology, the timely, personalized, and continuous delivery of nutritional support remains suboptimal in routine practice. A recent international survey among radiation oncologists from 65 countries confirmed that nutritional management practices remain heterogeneous, with persistent gaps in screening, referral pathways, and integration of nutritional care into routine oncology workflows [30]. Similar findings have been reported in previous surveys and real-world studies showing that nutritional care in oncology remains inconsistently implemented across settings despite its recognized clinical relevance [31,32,33,34,35].

Several barriers contributing to this gap have been identified, including organizational constraints, limited availability of specialized clinical nutrition professionals, and fragmentation between hospital-based care and community services [36]. These limitations may have important clinical, economic, and patient-centered consequences. In addition, insufficient nutritional guidance may place a significant burden on patients and caregivers. Nutrition is often a major concern for both patients and caregivers, particularly during active treatment and long-term follow-up [28,37]. When structured nutritional counselling and care are unavailable or inadequate, patients and caregivers may turn to informal or non-medical sources of information, including websites, social media, commercial programs, or unverified dietary advice. This may expose them to misleading or potentially harmful dietary regimens, including restrictive diets and approaches that claim to improve cancer outcomes without adequate scientific evidence [38]. In this context, digital health technologies may provide new opportunities to strengthen the delivery of nutritional care in oncology. Digital nutritional care can extend nutritional intervention and monitoring beyond hospital-based visits, facilitate early detection of nutritional deterioration or unintended weight changes, and provide patients and caregivers with timely, personalized, and reliable guidance in the home setting.

Previous systematic reviews have examined digital nutrition and lifestyle interventions in cancer populations and have included quality of life among the outcomes assessed [39,40]. However, their findings were limited by substantial heterogeneity in intervention content, delivery models, cancer populations, and outcome assessment, with quality of life evaluated only in a subset of the included studies. Since the completion of these reviews, additional original studies have been published, providing further evidence across active treatment, postoperative recovery, and survivorship settings.

Therefore, this narrative review builds on and updates the existing evidence by integrating more recent studies and examining broader dimensions of digital nutritional care beyond intervention effectiveness. In addition to nutritional and quality-of-life outcomes, we examine patient perspectives and the emerging role of caregivers, as well as professional supervision, clinical implementation, workflow integration, equity, digital burden, and potential risks associated with fully digital care pathways.

## 2. Material and Methods

This narrative review was based on a structured literature search conducted in PubMed/MEDLINE, Web of Science, and Scopus from database inception to 1 June 2026. The search was restricted to English-language publications. The search strategy was designed to address the main objectives of the review by combining a general search focused on digital nutritional care in oncology with two targeted searches addressing quality of life and patient and caregiver perspectives. It was developed for PubMed/MEDLINE and adapted to the syntax and indexing characteristics of Web of Science and Scopus. The complete database-specific search strategies are reported in the Supplementary Material File S1. The reference lists of relevant reviews and selected primary studies were also examined to identify additional pertinent publications. Following completion of the structured database searches, abstracts from major oncology meetings (American Society of Clinical Oncology [ASCO] Annual Meeting and European Society for Medical Oncology [ESMO] Congress) were additionally screened through a supplementary manual search performed later in June 2026 to identify emerging evidence not yet available as full-text publications.

Original studies, randomized trials, pilot and feasibility studies, surveys, and systematic or scoping reviews were considered eligible if they addressed digital nutritional care or digital lifestyle interventions with a nutritional component in patients with cancer, cancer survivors, or their caregivers.

For the purposes of this review, digital nutritional care was defined as any digital intervention designed to support nutritional assessment, monitoring, counselling, education, or nutrition-related self-management in oncology. Broader digital lifestyle or supportive care interventions were included only when they incorporated a clearly defined and identifiable nutritional component. Eligible interventions were therefore required to include at least one specific nutrition-related activity, such as dietary assessment, body weight monitoring, nutritional status assessment, weight management, or the management of nutrition impact symptoms.

Given the narrative design of the review, no formal independent screening, disagreement resolution procedures, quantitative record-flow reporting, or formal risk-of-bias assessment were performed. The restriction to English-language publications may have limited the identification of some relevant evidence. In addition, as study identification and selection were not conducted through a formal systematic-review process, some degree of selection bias cannot be excluded. These considerations should be taken into account when interpreting the findings of this review.

## 3. Digital Tools for Nutritional Care Delivery in Oncology

**Figure 1:**
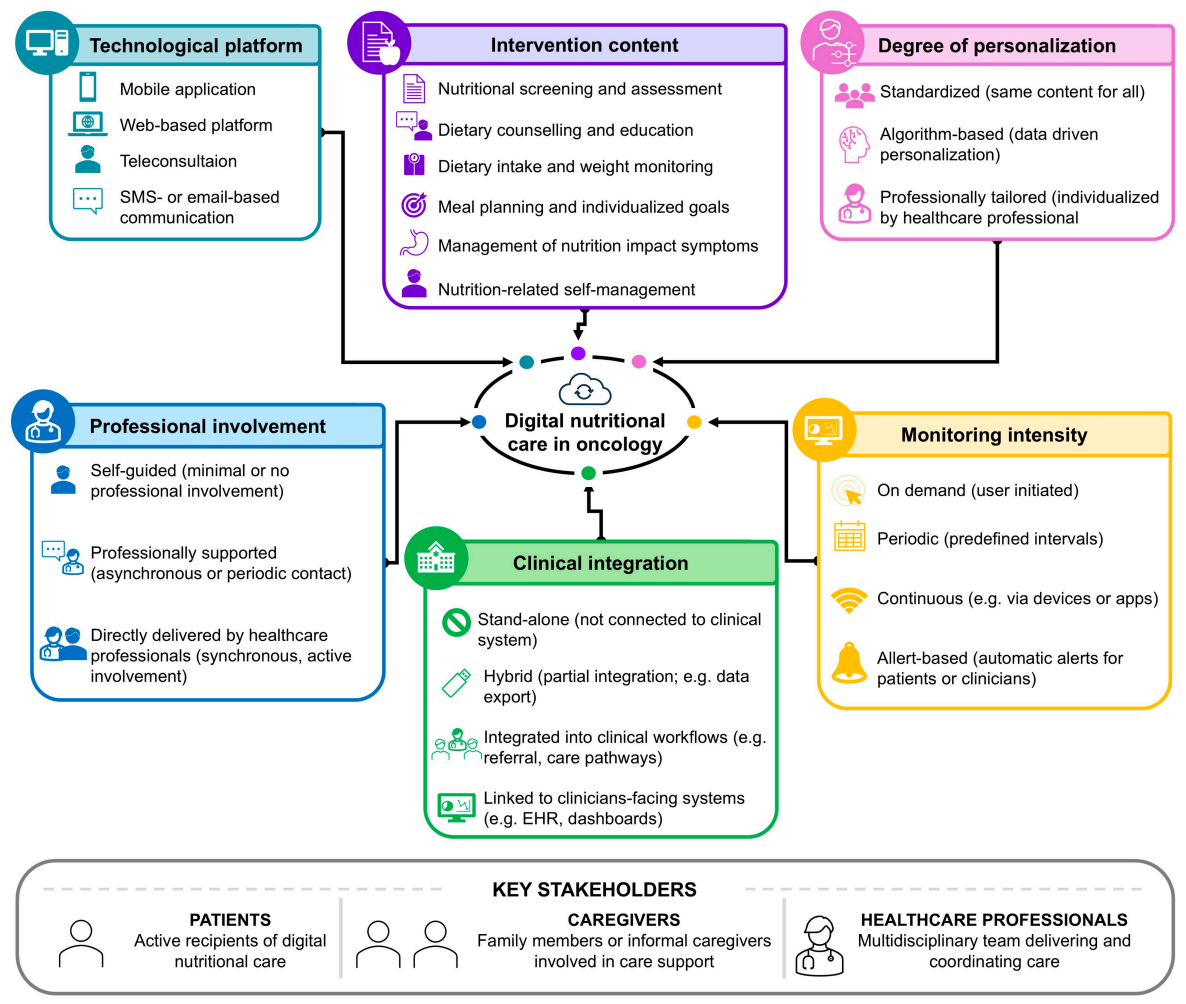
Multidimensional framework for digital nutritional care in oncology. Digital nutritional care interventions may be characterized across six complementary dimensions: technological platform, intervention content, degree of personalization, professional involvement, monitoring intensity, and clinical integration. Patients, caregivers, and healthcare professionals represent the key stakeholders involved throughout the digital nutritional care pathway. EHR: electronic health record; SMS: short message service.

Digital nutritional care can be broadly defined as the use of digital health technologies to deliver, facilitate, or enhance nutritional care across the cancer care continuum [41]. Rather than representing a single intervention or technology, digital nutritional care encompasses a range of approaches that can be characterized across several complementary dimensions, including technological platform, intervention content, degree of personalization, professional involvement, monitoring intensity, and integration into clinical workflows (Figure 1). These dimensions may be combined in different ways within individual interventions, while patients, caregivers, and healthcare professionals are the key stakeholders involved throughout the digital nutritional care pathway.

In oncology, this concept is particularly relevant in the home setting. Nutritional problems frequently develop or worsen outside the hospital and between scheduled visits, requiring patients and caregivers to manage dietary intake, symptoms, body weight changes, and treatment-related toxicities.

From a methodological perspective, digital nutritional care can be described according to three complementary dimensions: (i) the nutritional domains addressed, (ii) the mode of delivery, and (iii) the degree of personalization and clinical integration [39].

The nutritional domains commonly addressed through digital nutritional care tools include nutritional screening and risk assessment, monitoring of dietary intake and body weight, and management of nutrition impact symptoms [40]. Many interventions integrate nutritional components into broader supportive care or survivorship programs that also include behavioral strategies and physical activity [39,40,42].

The delivery of digital nutritional care can be broadly organized into four main models: mobile applications, web-based platforms, telemedicine-based interventions, and SMS- or email-based communication (Table 1).

Several studies combine digital nutritional care with additional forms of remote contact, including telephone calls [48,56], in-person consultations, group meetings, or training sessions [49,57], or a combination of remote and in-person contact [42]. This additional support is provided directly by healthcare professionals, including dietitians, nutrition specialists, nurses, and trained coaches, thereby adding human and clinical components to digital intervention. An additional aspect of innovation involves linking patient-generated data to clinician-facing interfaces [58]. Mobile applications and web-based platforms may allow patients to report dietary intake, body weight, symptoms, physical activity, treatment-related toxicities, and quality-of-life outcomes in real time or at predefined intervals. However, the clinical relevance of these data depends on whether they are accessible, interpretable, and actionable by the healthcare team. In this regard, clinician-facing dashboards may represent a key component of more advanced digital nutritional care models, enabling healthcare professionals to review longitudinal trends, identify early signs of nutritional deterioration or unintended weight gain, prioritize patients requiring intervention, and provide timely feedback. From a practical perspective, different modes of delivering digital nutritional care should not be considered equivalent. Teleconsultation involves direct interaction with a healthcare professional and therefore generally allows for a higher degree of personalization. A similar level of personalization may also be achieved through other digital tools, such as mobile applications, web-based platforms, or SMS- or email-based interventions, when combined with human-mediated contact or professional feedback. Nevertheless, these hybrid models do not fully address the limited availability of dedicated clinical nutrition professionals. For this reason, emerging technological developments are increasingly oriented toward more scalable forms of personalized nutritional guidance, based either on predefined algorithms [45,59,60] or, in the near future, on more advanced artificial intelligence-supported systems.

The use of large language models (LLMs) to support patients with cancer and their caregivers is rapidly expanding [61]. In patients with breast cancer, LLM-generated meal plans and grocery lists have been compared with those developed by dietitians, showing similarities in macronutrient composition under predefined prompting conditions [62]. LLM-generated outputs have also been evaluated in patients with head and neck cancer for their ability to translate guideline-based recommendations on nutrition impact symptoms into more accessible, patient-oriented advice [63].

Beyond stand-alone LLM-based tools, agentic artificial intelligence (AI) systems may support more complex and longitudinal nutritional care processes by combining generative models with planning capabilities, memory, external data sources, and predefined clinical rules [64]. In nutritional oncology, LLMs may answer nutrition-related questions, generate symptom management advice, or suggest meal plans and grocery lists, whereas agentic systems may support more complex, multistep nutritional care processes. As the volume and complexity of patient-generated and clinical data increase, more advanced AI-supported approaches may help integrate multiple sources of information and provide a more comprehensive overview of patients’ evolving nutritional needs. Their outputs could be incorporated into clinical decision support systems, while final interpretation and oversight remain the responsibility of healthcare professionals. Human supervision is essential to ensure that digital care preserves clinical judgement, respects patient autonomy and dignity, and remains focused on outcomes that matter most to patients, including quality of life. Nevertheless, their application in cancer-related nutritional care remains at an early stage. Several limitations and risks must be addressed before these systems can be integrated into routine practice. Current evidence is largely based on proof-of-concept studies with limited external validity, and their clinical appropriateness, safety, acceptability, adherence, and effects on patient outcomes remain uncertain. LLM- and agentic AI-based systems may generate inaccurate or fabricated recommendations, suggest inappropriate dietary restrictions, or fail to adequately account for comorbidities, treatment-related toxicities, drug–nutrient interactions, swallowing difficulties, metabolic alterations, and individual nutritional requirements. Additional concerns include privacy, cybersecurity, algorithmic bias, transparency, accountability, and evolving regulatory requirements. Their clinical development should therefore incorporate validated knowledge sources, predefined safety constraints, secure data governance, prospective evaluation, and clear escalation pathways to qualified nutrition professionals.

## 4. From Digital Tools to Integrated Care Pathways

The clinical value of digital nutritional care depends not only on the availability of technological tools but also on their effective integration into oncology care pathways. Digital health and telehealth interventions are increasingly used across the cancer care continuum, but their impact is shaped by implementation quality, workflow integration, professional engagement, and patient-centered delivery [41,65]. Patient-generated nutritional data become clinically meaningful only when they are reviewed, interpreted, and translated into appropriate clinical actions. For this reason, implementation should extend beyond the simple adoption of digital tools to include the organization of care. Digital nutritional pathways should define which data are collected, how frequently monitoring occurs, who is responsible for reviewing the information, and which thresholds trigger professional intervention. This is particularly important because integrating patient-generated health data into clinical practice and electronic health records remains a recognized challenge, requiring attention to data quality, interpretability, usability, clinician workload, and interoperability [66,67]. Sustainable implementation also requires appropriate reimbursement and funding models. In many healthcare systems, reimbursement pathways for teleconsultations, remote nutritional monitoring, digital platform maintenance, and professional review of patient-generated data remain inconsistent or insufficiently defined. Without adequate financial and organizational support, digital nutritional care may remain confined to research projects or locally funded initiatives rather than becoming part of routine oncology care. A clinically integrated model should also clearly define the roles of the multidisciplinary team. Dietitians and nutrition specialists remain central to the assessment, counselling, and management of complex nutritional problems, while oncologists, nurses, rehabilitation professionals, psychologists, and caregivers may contribute to early identification of needs, symptom management, behavioral support, and continuity of care [68]. Digital tools may facilitate this coordination by making relevant information more visible and improving communication among hospital services, community care providers, patients, and families. Recent work on implementation priorities for digital health in cancer care similarly emphasizes the need to align digital tools with real-world care processes, stakeholder needs, and service capacity [69].

## 5. Current Evidence on Digital Nutritional Interventions and Quality of Life

The added value of digital nutritional care also depends on its ability to improve nutritional care delivery and translate into patient-centered benefits, including quality of life. Digital nutritional care and combined digital lifestyle interventions may influence quality of life through several mechanisms, including improvements in nutritional status, earlier management of nutrition impact symptoms, prevention of unintended weight loss or weight gain, enhanced self-efficacy, greater continuity of care, and better support for patients and caregivers in the home setting. Available studies evaluating digital nutritional and lifestyle interventions in oncology are summarized in Table 2.

Recent original studies reported mixed quality-of-life outcomes across cancer settings. In postoperative patients with gastric cancer, the 12-week nurse-led WeChat-based nutritional intervention did not improve global quality of life but was associated with more favorable nutritional outcomes. The Patient-Generated Subjective Global Assessment (PG-SGA) score increased significantly in the control group from baseline to the first follow-up, whereas the reduction from the first to the second follow-up was greater within the intervention group. In parallel, the intervention group showed a more favorable change in the percentage weight loss and higher energy intake than the control group [42]. By contrast, a 12-month personalized digital exercise and nutrition rehabilitation program in postoperative patients with gastric cancer did not significantly improve either quality of life or changes in body weight compared with standard rehabilitation [45].

Similarly, in non-small cell lung cancer survivors, a 12-week lifestyle application combining nutrition, physical activity, and breathing and relaxation modules did not significantly improve global quality of life, as assessed using the EORTC QLQ-C30. Exercise capacity improved in the intervention group, whereas BMI, risk of low protein intake, and appetite remained stable in both groups [43]. A telehealth lifestyle intervention in breast cancer survivors also did not result in a significant change in quality of life or produce a consistent favorable change in body weight, although waist circumference decreased during follow-up [53]. A pilot randomized trial evaluated a closed-loop chatbot integrated into usual care for patients with breast or colorectal cancer receiving chemotherapy. Global health status improved significantly within the chatbot group. Cancer-related fatigue remained stable in the chatbot group but worsened with usual care, resulting in a significant between-group difference [60]. Among the 331 content-specific patient queries recorded during the study, nutrition-related requests represented the second most frequent category after cancer-related knowledge [60].

Favorable results were observed in interventions combining sustained behavioral support with weight management. In breast cancer survivors, a 12-month remotely delivered program based on telephone counselling, mailed materials, and optional text messages resulted in a statistically significant between-group improvement in the Short Form-36 physical quality-of-life score compared with usual care (between-group difference, 2.7 points; 95% CI, 0.7–4.6; *p* = 0.007), alongside greater improvements in body weight and metabolic risk [56]. Likewise, in a three-arm trial involving endometrial cancer survivors with obesity, quality-of-life outcomes improved over time. In the between-group comparison, telemedicine resulted in significantly greater improvements in the SF-12 Physical Health Component Score and cancer-related body image than text-based support [54]. Reductions in body weight, percentage total weight loss, and body circumferences were observed across all three groups, with no significant between-group differences [54].

In a large primary-care survivorship trial involving prostate, colorectal, and breast cancer survivors, quality of life improved over 6 months within all three digital support groups. However, no significant between-group differences were observed [50].

Conversely, among prostate cancer survivors, progressive intensification of web-based behavioral support, including personalized dietary and exercise prescriptions, wearable monitoring, text messages, and coaching calls, did not improve quality of life [51].

Recent conference reports have described emerging findings on telephone-based and collaborative remote care. In the BWEL trial (NCT02750826) [70], a 24-month telephone-based intervention combining dietary modification, physical activity, and behavioral support achieved clinically meaningful weight loss in women with stage II–III breast cancer and overweight or obesity [71]. Recent conference data also suggest improvements in physical function and global physical and mental health, as well as reductions in fatigue, which were generally maintained at 24 months [71,72]. In the ME-DEA trial (NCT04304924) [73], a personalized telephone-based weight-loss intervention did not significantly improve cancer-related fatigue at 12 months but resulted in significantly greater weight loss among breast cancer survivors with overweight or obesity [74]. Finally, the T-NICE trial (NCT06332664) [75] reported that collaborative telenutritional care reduced the incidence of cachexia and improved quality of life and nutritional status compared with standard nutritional care in patients with stage IV gastrointestinal cancer receiving chemotherapy [76]. Although these findings remain preliminary pending confirmation in full peer-reviewed publications, they indicate that the evidence base on digital nutritional care is likely to expand in the coming years.

Some interventions combining digital tools with personalization, professional feedback, behavioral support, or clinically actionable monitoring reported favorable patient-centered outcomes. However, heterogeneity across studies limits the interpretation of these findings. It should also be acknowledged that statistical significance does not necessarily imply clinical relevance. These observations should therefore be considered hypothesis-generating rather than evidence of comparative effectiveness. Moreover, several of the included interventions were multimodal, combining nutrition with physical activity, symptom management, behavioral support, psychological care, or broader survivorship components. Consequently, any observed effect on quality of life should be interpreted as arising from the intervention as a whole and cannot be attributed specifically to its nutritional component.

The overall quality of the available evidence warrants cautious interpretation of the current findings. Many studies are small, single-center pilot, feasibility, or exploratory investigations, and quality of life is frequently assessed as a secondary or exploratory outcome. These limitations reduce the certainty and generalizability of the current findings and highlight the need for adequately powered multicenter studies with appropriate follow-up and quality of life included as a prespecified primary outcome.

An important limitation of the current evidence is that quality of life has not been systematically assessed across digital nutritional interventions. Several recent studies have primarily focused on nutritional status, dietary behavior, body composition, feasibility, or delivery of nutritional treatment, without including quality of life as a prespecified outcome. For instance, telehealth-based nutritional care in patients with hepatocellular or colorectal cancer at nutritional risk, as well as a WeChat-based nutritional program in patients with ovarian cancer undergoing chemotherapy, improved nutritional status but did not assess quality of life [46,55]. Similarly, an individualized WeChat-based intervention for patients after gastrectomy improved nutrition-related behaviors [57], whereas a web-based intervention in patients with breast cancer [52] and an intervention involving cancer survivor–partner dyads [47] reported improvements in body composition and dietary quality without evaluating quality of life. A hybrid intervention combining group sessions, text- or email-based newsletters, and website access also improved diet quality and exercise time in breast cancer survivors but did not include quality of life among its reported outcomes [49].

This limitation is clinically relevant because digital nutritional care is intended to support patients in their daily lives. Future studies should therefore more consistently incorporate validated patient-reported outcomes, with quality of life assessed alongside nutritional and clinical outcomes. Beyond quality of life, digital nutritional and lifestyle interventions have primarily been evaluated in terms of short-term nutritional, behavioral, functional, and metabolic outcomes. However, most studies have relatively limited follow-up periods, and it remains unclear whether the observed benefits are sustained over time or translate into clinically meaningful long-term outcomes, such as improved treatment adherence and tolerance, fewer treatment interruptions, reduced hospitalizations or readmissions, lower cancer recurrence, and improved survival.

Previous systematic reviews (Table 3) also highlight the limited and inconsistent assessment of quality of life in this field. Kiss et al. included 16 studies of technology-supported nutrition and physical activity interventions in adults with cancer, but only 9 assessed quality of life; among these, improvement was reported in 4 studies [39]. Similarly, Ng et al. identified 13 studies of mobile app-based nutritional interventions, of which only 6 evaluated quality of life and only 2 reported a significant benefit [40]. Therefore, beyond the heterogeneity of intervention effects, the underrepresentation of quality of life as a prespecified outcome remains a major methodological weakness of the current evidence base.

## 6. Patient Perspectives and the Emerging Role of Caregivers in Digital Nutritional Care

Current evidence suggests that digital nutritional and lifestyle interventions in oncology are generally feasible, although their clinical effects remain heterogeneous. Across different cancer populations and care settings, patients have successfully engaged with mobile applications, web-based platforms, teleconsultations, SMS- or email-based communication, WeChat-based tools, and hybrid models combining digital support with professional feedback or in-person training. These findings support the practical applicability of digital nutritional care during active treatment, postoperative recovery, and survivorship. However, feasibility alone is insufficient to establish the value of these interventions. In routine oncology care, digital nutritional support is likely to be effective only if patients and caregivers perceive it as acceptable, usable, and relevant to their daily needs.

Survey data have shown that nutritional problems in cancer care are frequently underrecognized and undertreated, despite substantial patient-reported needs for nutritional information and support [77]. More recent survey evidence also indicates that patients commonly seek information on nutrition and physical activity from multiple sources, highlighting the importance of providing reliable, accessible, and clinically appropriate guidance. In this context, digital nutritional care may offer a useful channel to improve access to information and support, provided that the content is understandable, trustworthy, and integrated with professional care.

Available evidence on patient preferences suggests that digital delivery should not be interpreted as a replacement for human interaction. In young adult cancer survivors, a survey on preferences for diet and physical activity interventions showed interest in technology-supported strategies but also highlighted the value of one-to-one telehealth dietary counselling, virtual cooking classes, and personal contact in the development of lifestyle interventions [78]. Similarly, broader evidence on perspectives of patients with cancer regarding digital health technologies indicates that they value tools that are easy to use, personalized, supportive of self-management, and able to facilitate communication with healthcare professionals [79]. These findings are consistent with the broader digital health literature, which identifies empowerment, personalization, and integration with care as important determinants of patient engagement with digital tools [80].

Caregiver involvement represents an additional and still underexplored dimension of digital nutritional care. A mixed-methods study aimed at developing a mobile application for caregivers of patients with head and neck cancer identified nutritional recovery as a relevant area of support and incorporated educational information, caregiving tips, intake tracking, support videos, and resources into a caregiver-facing digital intervention [81]. A subsequent evaluation of the Healthy Eating and Recovery Together intervention, designed to support head and neck cancer survivors and caregivers, showed favorable feasibility and acceptability of a digital health approach combining needs assessment, a tailored care plan, and caregiver application [82]. Despite the central role of caregivers in the daily management of cancer-related nutritional problems, their participation in digital nutritional intervention studies remains limited. Most available studies have focused primarily on feasibility and acceptability rather than on caregiver burden, confidence in managing nutrition impact symptoms, unmet informational needs, anxiety, usability, or the time demands related to digital monitoring. Future studies should therefore include caregiver-specific outcomes and evaluate whether digital support reduces uncertainty and burden.

Patient- and caregiver-centered digital nutritional care should be designed around practical needs rather than technology alone. Future development should therefore prioritize user-centered design, caregiver involvement, professional oversight, and integration with routine care pathways.

## 7. The Risk of Going Fully Digital

Although digital nutritional care may improve access, continuity, and personalization of care, fully digital models also raise relevant clinical, ethical, and organizational concerns.

The first concern is the progressive loss of human contact. Nutritional problems in cancer are often associated with fear, fatigue, and emotional distress. Standardized digital messages may be insufficient when patients or caregivers require reassurance, clarification, or individualized discussion.

A second concern is digital burden. Repeated food logging, symptom reporting, weight monitoring, reminders, and engagement with educational modules may support self-management but may also become demanding during active treatment. Caregivers may experience additional workload when expected to assist with data entry, monitoring, or interpretation of app-based feedback. Digital nutritional tools should therefore minimize cognitive load and collect only data that are clinically meaningful and actionable.

Food tracking warrants particular caution. Dietary self-monitoring may help identify inadequate intake and guide counselling, but evidence from the broader digital health and eating behavior literature suggests that diet and fitness apps may contribute to psychological distress or maladaptive eating behaviors in vulnerable individuals, particularly when they rely on calorie counting, rigid goals, persuasive feedback, or continuous self-surveillance [83,84,85]. In oncology, food tracking should therefore be used proportionately and preferably under professional supervision.

Equity is another major concern. Digital health may improve access for patients living far from specialist services but may also widen inequalities among older adults and individuals with low digital literacy, cognitive impairment, or limited internet access. Recent work on telehealth accessibility in cancer care has shown that digital divides may persist across geographic and sociodemographic contexts [86].

Digital nutritional technologies may collect sensitive information. Patients should therefore receive clear information on which data are collected, how they are stored and used, who can access them, and whether they may be shared with third parties. Secure authentication, encryption, access controls, audit trails, and appropriate procedures for managing data breaches are particularly important when patient-generated data are integrated with electronic health records or clinician-facing platforms [87]. Ethical concerns also include informed consent, transparency, patient autonomy, algorithmic bias, and accountability when recommendations are generated or prioritized by automated systems [88]. Clear professional oversight and escalation pathways are therefore required to ensure that digital support remains safe and clinically appropriate.

Nutritional care in oncology is not limited to delivering dietary information or collecting food-intake data. It also requires clinical interpretation, empathic communication, shared decision-making, motivational support, and adaptation of recommendations to symptoms, treatment phase, prognosis, psychosocial context, and patient preferences. Therefore, digital tools should be regarded as facilitators of nutritional care delivery rather than complete substitutes for professional interaction [41].

## 8. Conclusions

Digital nutritional care may help address a major unmet need in oncology by extending timely, personalized, and continuous nutritional support beyond conventional clinical encounters. Overall, the available evidence suggests that digital nutritional and lifestyle interventions are feasible and may improve nutritional status, dietary behaviors, weight-related outcomes, and physical activity. However, their effects on quality of life remain variable, and substantial heterogeneity in intervention design, duration, professional involvement, personalization, clinical integration, and overall evidence quality limits the interpretation of the available findings. Importantly, quality of life has not been consistently included as an outcome in many studies, despite being a central patient-centered endpoint for interventions intended to support daily nutritional management.

Current evidence also indicates that patients value reliable, accessible, and personalized digital support, particularly when it facilitates communication with healthcare professionals. Caregiver-specific evidence remains limited and should therefore be regarded as an emerging component of this field. Future digital nutritional care should not evolve toward a complete replacement of conventional counselling but rather toward hybrid and supervised models that preserve human contact, clinical judgement, and caregiver involvement. When developed with attention to usability, equity, psychological safety, and meaningful integration into oncology care pathways, digital tools may become an important component of supportive oncology rather than an additional burden for patients and families.

## Figures and Tables

**Table 1:** Main delivery modes for digital nutritional care.

Delivery Mode	Description and Main Components	References
Mobile applications	Educational content, self-monitoring tools, reminders, dietary and lifestyle tracking, and feedback.	[40,43,44,45,46]
Web-based platforms	Interventions delivered through online platforms or software, including educational modules, lifestyle resources, self-management tools, and interactive content.	[39,47,48,49,50,51,52]
Teleconsultation	Nutritional or lifestyle support delivered remotely through video consultations, usually involving a healthcare professional.	[48,53,54,55]
SMS- or email-based communication	Periodic delivery of nutritional or lifestyle content through SMS or email, including educational messages, reminders, behavioral prompts, and reinforcement of healthy eating and physical activity recommendations between scheduled contacts.	[42,48,49,54,56,57]

SMS: Short Message Service.

**Table 2:** Primary intervention studies evaluating digital nutritional care and multimodal lifestyle interventions, with a focus on quality-of-life outcomes in oncology.

Population and Sample Size	Study Design and Duration	Intervention	Comparator	QoL Instrument and Outcome Status	QoL Findings	Additional Findings	Reference
Postoperative patients with gastric cancer; *N* = 257	RCT, 2:1 allocation; 12 months	Mobile health intervention with algorithm-based personalization and dietary tracking based on estimated nutritional requirements	Standard rehabilitation	EORTC QLQ-C30; secondary outcome	No significant between-group differences in EORTC QLQ-C30 global health status or functional domains	-	[45]
Postoperative patients with gastric cancer; *N* = 108	RCT; 12 weeks	WeChat-based mobile health intervention including initial face-to-face education, biweekly telephone calls, nutritional status reporting, dietary goals, meal plans, food and weight diaries, peer communication, symptom management guidance, and consultation	Usual care	EORTC QLQ-C30; secondary outcome	No significant differences in EORTC QLQ-C30 global quality of life	Improvements in PG-SGA scores, lower weight loss, higher energy and proteins intake in the intervention group	[42]
NSCLC survivors; *N* = 18	RCT; 12 weeks	Lifestyle mobile application including nutrition and weekly educational content delivered through podcasts and readings	Standard care	EORTC QLQ-C30; primary outcome	No significant between-group difference (adjusted mean difference: 2.89, 95% CI: –17.76–23.53, *p* = 0.77)	Increased exercise capacity in the intervention group	[43]
Patients who had completed primary treatment for prostate, colorectal, or breast cancer; *N* = 1809	RCT, 1:1:1 allocation; 6 months	Generic digital support (“LiveWell”) based on an online software; Tailored digital support (“Renewed”), including symptom management, physical activity, diet, weight loss, and distress components; “Renewed plus”: “Renewed” with brief email and telephone support	Comparison among the three digital support modalities	EORTC QLQ-C30; primary outcome	QoL improved in all three groups at 6 months, with no significant between-group differences.	-	[50]
Patients with breast and colorectal cancer receiving chemotherapy; *N* = 40	Pilot RCT; 12 weeks	Mobile- and web-based closed-loop chatbot intervention, including oncology nutrition plus usual care	Usual care	EORTC QLQ-C30; secondary outcome	Global health status improved in the chatbot group (*p* = 0.04), while physical functioning remained stable. Fatigue remained stable with the chatbot but worsened with usual care, with a significant between-group difference (*p* = 0.02)	-	[60]
Breast cancer survivors; *N* = 34	Single-arm study; 14 weeks	Telehealth intervention including healthy eating recommendations, fruit and vegetable intake recording, and weekly 24-h dietary recalls	No comparator	EORTC QLQ-C30; secondary outcome	No statistically significant change	Increased exercise time	[53]
Breast cancer survivors; *N* = 159	RCT; 12 months	Telephone-based weight loss intervention including 22 calls, mailed materials, optional text messages, dietary counselling, and physical activity support	Usual care	SF-36; secondary outcome	At 12 months, the intervention significantly improved physical quality of life compared with usual care (between-group difference 2.7 points, 95% CI 0.7–4.6; *p* = 0.007; Cohen’s d = 0.40)	Greater improvements in weight, fat mass, metabolic syndrome risk score, waist circumference and fasting plasma glucose	[56]
Endometrial cancer survivors with obesity; *N* = 196	RCT, 1:1:1 allocation 6 months	Telemedicine with Wi-Fi scales; Text-based dietary support, and messaging; Enhanced usual care	Comparison among telemedicine, text-based support, and enhanced usual care	SF-12; secondary outcome	Psychosocial outcomes generally improved across all groups. Compared with the text-messaging arm, telemedicine significantly improved the SF-12 Physical Health Component Score and cancer-related body image. No between-group differences were observed for relationship functioning or depressive symptoms.	Physical activity improvements in enhanced nutritional care group	[54]
Prostate cancer survivors; *N* = 202	RCT, 1:1:1:1 allocation; 3 months	Increasing levels of web-based behavioral support: Level 1: website alone; Level 2: Level 1 + personalized diet and exercise prescription; Level 3: Level 2 + Fitbit and text messages; Level 4: Level 3 + coaching calls	Comparison across four levels of increasing web-based behavioral support, with Level 1 (website only) as the reference group	EORTC QLQ-C30 and PROMIS Fatigue; secondary/exploratory outcomes	No significant QoL benefit. Level 3 showed a borderline improvement in Global Health versus Level 1 (+5.46; 95% CI −0.02 to 10.95).	-	[51]

RCT: randomized controlled trial; NSCLC: non-small cell lung cancer; QoL: quality of life; EORTC QLQ-C30: European Organisation for Research and Treatment of Cancer Quality of Life Questionnaire Core 30; PG-SGA: Patient-Generated Subjective Global Assessment; PROMIS: Patient-Reported Outcomes Measurement Information System; SF-12: 12-Item Short Form Health Survey; SF-36: 36-Item Short Form Health Survey; CI: Confidence Interval. The table includes only primary studies published after the search periods of the systematic reviews by Kiss et al. [39] and Ng et al. [40] (Table 3), to avoid overlap and double counting of evidence.

**Table 3:** Previous systematic reviews of digital nutritional and lifestyle interventions in oncology.

Population and Sample Size	Intervention	Effects on QoL	Additional Findings	Reference
Patients with cancer *N* = 16 studies of which 9 evaluated QoL	Web-based platforms and apps including nutrition and physical activity components	↑ QoL in 4/9 studies	↑ 8/15 studies physical activity behavior ↑ 2/7 studies dietary behavior	[39]
Patients with cancer *N* = 13 studies of which 6 evaluated QoL	Mobile app-based nutritional interventions	↑ QoL 2/6 studies	↑ nutritional status	[40]

↑: improved.

## Data Availability

No new data were created or analyzed in this study. Data sharing is not applicable to this article.

## Supplementary Materials

The following supporting information can be downloaded at https://www.mdpi.com/article/10.3390/cancers18152468/s1, File S1: Database-specific literature search strategies.

## Abbreviations

The following abbreviations are used in this manuscript:

SMS	Short Message Service
LLM	large language model
RCT	randomized controlled trial
NSCLC	non-small cell lung cancer
QoL	quality of life
EORTC QLQ-C30	European Organisation for Research and Treatment of Cancer Quality of Life Questionnaire Core 30
SF-36	Short Form-36
PG-SGA	Patient-Generated Subjective Global Assessment
