# Supplementary material for: Digital Nutritional Care in Oncology: Opportunities to Enhance Quality of Life and Support Patient-Centered Care
Source: Cancers (Basel). 2026 Jul 31;18(15):2468. doi: 10.3390/cancers18152468 (PMC13465412; doi:10.3390/cancers18152468)
Supplement: Supplementary file 1 [file cancers-18-02468-s001.zip › cancers-4436315-supplementary.pdf]

File S1. Database-specific literature search strategies

The searches were conducted from database inception to 1 June 2026 and were restricted to English-language publications. Terms within each conceptual domain were combined using the Boolean operator OR, whereas the different conceptual domains were combined using AND.

PubMed/MEDLINE

Search 1. General search on digital nutritional care in oncology

(cancer OR oncology) AND (nutrition OR "nutritional care" OR "nutritional support" OR diet OR "dietary intervention" OR "lifestyle intervention" OR "physical activity") AND ("digital health" OR eHealth OR mHealth OR telehealth OR telemedicine OR "mobile application" OR "web-based intervention" OR "remote monitoring" OR "text messaging" OR WeChat OR chatbot)

Search 2. Digital nutritional care and quality of life

(cancer OR oncology) AND (nutrition OR "nutritional care" OR "nutritional support" OR diet OR "dietary intervention" OR "lifestyle intervention" OR "physical activity") AND ("digital health" OR eHealth OR mHealth OR telehealth OR telemedicine OR "mobile application" OR "web-based intervention" OR "remote monitoring" OR "text messaging" OR WeChat OR chatbot) AND ("quality of life" OR "health-related quality of life" OR QoL OR HRQoL OR "patient-reported outcomes" OR "global health status")

Search 3. Digital nutritional care and patient or caregiver perspectives

(cancer OR oncology) AND (nutrition OR "nutritional care" OR "nutritional support" OR diet OR "dietary intervention" OR "lifestyle intervention" OR "physical activity") AND ("digital health" OR eHealth OR mHealth OR telehealth OR telemedicine OR "mobile application" OR "web-based intervention" OR "remote monitoring" OR "text messaging" OR WeChat OR chatbot) AND ("patient experience" OR "patient perspectives" OR caregiver OR acceptability OR usability OR feasibility)

Web of Science Core Collection

TS indicates Topic Search.

Search 1. General search on digital nutritional care in oncology

TS=(cancer OR oncology) AND TS=(nutrition OR “nutritional care” OR “nutritional support” OR diet OR “dietary intervention” OR “lifestyle intervention” OR “physical activity”) AND TS=(“digital health” OR eHealth OR mHealth OR telehealth OR telemedicine OR “mobile application” OR “web-based intervention” OR “remote monitoring” OR “text messaging” OR WeChat OR chatbot)

Search 2. Digital nutritional care and quality of life

TS=(cancer OR oncology) AND TS=(nutrition OR “nutritional care” OR “nutritional support” OR diet OR “dietary intervention” OR “lifestyle intervention” OR “physical activity”) AND TS=(“digital health” OR eHealth OR mHealth OR telehealth OR telemedicine OR “mobile application” OR “web-based intervention” OR “remote monitoring” OR “text messaging” OR WeChat OR chatbot) AND TS=(“quality of life” OR “health-related quality of life” OR QoL OR HRQoL OR “patient-reported outcomes” OR “global health status”)

Search 3. Digital nutritional care and patient or caregiver perspectives

TS=(cancer OR oncology) AND TS=(nutrition OR “nutritional care” OR “nutritional support” OR diet OR “dietary intervention” OR “lifestyle intervention” OR “physical activity”) AND TS=(“digital health” OR eHealth OR mHealth OR telehealth OR telemedicine OR “mobile application” OR “web-based intervention” OR “remote monitoring” OR “text messaging” OR WeChat OR chatbot) AND TS=(“patient experience” OR “patient perspectives” OR caregiver OR acceptability OR usability OR feasibility)

Scopus

TITLE-ABS-KEY indicates a search within the article title, abstract, and keywords.

Search 1. General search on digital nutritional care in oncology

TITLE-ABS-KEY(cancer OR oncology) AND TITLE-ABS-KEY(nutrition OR "nutritional care" OR "nutritional support" OR diet OR "dietary intervention" OR "lifestyle intervention" OR "physical activity") AND TITLE-ABS-KEY("digital health" OR eHealth OR mHealth OR telehealth OR telemedicine OR "mobile application" OR "web-based intervention" OR "remote monitoring" OR "text messaging" OR WeChat OR chatbot)

Search 2. Digital nutritional care and quality of life

TITLE-ABS-KEY(cancer OR oncology) AND TITLE-ABS-KEY(nutrition OR "nutritional care" OR "nutritional support" OR diet OR "dietary intervention" OR "lifestyle intervention" OR "physical activity") AND TITLE-ABS-KEY("digital health" OR eHealth OR mHealth OR telehealth OR telemedicine OR "mobile application" OR "web-based intervention" OR "remote monitoring" OR "text messaging" OR WeChat OR chatbot) AND TITLE-ABS-KEY("quality of life" OR "health-related quality of life" OR QoL OR HRQoL OR "patient-reported outcomes" OR "global health status")

Search 3. Digital nutritional care and patient or caregiver perspectives

TITLE-ABS-KEY(cancer OR oncology) AND TITLE-ABS-KEY(nutrition OR "nutritional care" OR "nutritional support" OR diet OR "dietary intervention" OR "lifestyle intervention" OR "physical activity") AND TITLE-ABS-KEY("digital health" OR eHealth OR mHealth OR telehealth OR telemedicine OR "mobile application" OR "web-based intervention" OR "remote monitoring" OR "text messaging" OR WeChat OR chatbot) AND TITLE-ABS-KEY("patient experience" OR "patient perspectives" OR caregiver OR acceptability OR usability OR feasibility)
